# HIV restriction in quiescent CD4^+^ T cells

**DOI:** 10.1186/1742-4690-10-37

**Published:** 2013-04-04

**Authors:** Jerome A Zack, Sohn G Kim, Dimitrios N Vatakis

**Affiliations:** 1 Department of Medicine, Division of Hematology-Oncology, David Geffen School of Medicine at UCLA, Los Angeles, CA, 90095, USA; 2 Department of Microbiology, Immunology and Molecular Genetics, David Geffen School of Medicine at UCLA, Los Angeles, CA, 90095, USA; 3 UCLA AIDS Institute, David Geffen School of Medicine at UCLA, Los Angeles, CA, 90095, USA

**Keywords:** Quiescent CD4 T cells, HIV infection, Host restriction factors

## Abstract

The restriction of the Human Immunodeficiency Virus (HIV) infection in quiescent CD4^+^ T cells has been an area of active investigation. Early studies have suggested that this T cell subset is refractory to infection by the virus. Subsequently it was demonstrated that quiescent cells could be infected at low levels; nevertheless these observations supported the earlier assertions of debilitating defects in the viral life cycle. This phenomenon raised hopes that identification of the block in quiescent cells could lead to the development of new therapies against HIV. As limiting levels of raw cellular factors such as nucleotides did not account for the block to infection, a number of groups pursued the identification of cellular proteins whose presence or absence may impact the permissiveness of quiescent T cells to HIV infection. A series of studies in the past few years have identified a number of host factors implicated in the block to infection. In this review, we will present the progress made, other avenues of investigation and the potential impact these studies have in the development of more effective therapies against HIV.

## Review

### Introduction

Quiescence is a unique feature of our immune system as T lymphocytes can remain at a non-dividing state for prolonged periods of time. The majority of circulating T cells in blood are in a quiescent state. This is characterized by low metabolic rates, low levels of transcription, small size and very long periods of survival [1,2]. It was long thought that T cell quiescence was a default state. A recent series of studies reversed this notion as they demonstrated that a number of transcription factors actively maintained this state [1-10]. To this date, LKLF [3,4,8], FOXO1,3 and 4 [7,11-18], and Tob [6,10,19,20] have been identified as key factors that maintain T cell quiescence . Loss of expression of any of the above proteins resulted in aberrant T cell proliferation, cellular damage due to higher metabolism, and cell death. CD4^+^ T cell quiescence and its effect on HIV infection has been a topic of intense investigation as early studies indicated that they are resistant to HIV infection. As a result a strong interest was developed to identify cellular factors that mediate this block and can potentially be the basis for effective therapeutic approaches against HIV. None of the factors regulating T cell quiescence have been implicated in influencing HIV infection.

In this review, we will discuss the steps of the viral life cycle inhibited in quiescent CD4^+^ T cells, the factors involved and the impact these studies have in understanding HIV infection in quiescent T cells as well as the development of better targets against the virus.

### HIV replication is defective in quiescent CD4^+^ T cells

For the past two decades, the infection of quiescent CD4 T cells by HIV has been an area of intense investigation. Unlike other retroviruses, HIV replication is not dependent on cell cycle. Nevertheless, HIV and other lentiviruses more efficiently infect non-dividing cells and establish a latent infection [21-23]. While early reports supported the notion that only pre-activated T cells can be infected by HIV [24-26], subsequent studies showed that quiescent T cells could be infected by the virus [27-30]. Yet, key differences arose relating to the degree and levels of infection efficiency.

On the one hand it was shown that HIV viral entry and initiation of reverse transcription were not affected. However, completion of reverse transcription was inefficient resulting in the accumulation of labile, intermediate viral cDNA species [28,29]. Rescue of infection was possible with stimulation but it was temporally sensitive as production of viral progeny decreased at later reactivation timepoints [29]. Additional work focusing on the CD25- (non-activated) and CD25+ (activated) T cell populations lent more support to the notion that quiescent T cells are resistant to HIV infection [31-33]. In the absence of any stimulation, HIV infection of CD25- T cells failed while that of CD25+ was successful. Furthermore, when total human peripheral blood monocytes were infected, the CD25- population did carry viral cDNA suggesting either bystander activation of the non-activated population or more efficient infection via cell-cell contact. Finally, Tang and colleagues further supported the above observations by demonstrating that infection of quiescent cells with HIV did not result in the production of virus [34].

On the other hand, other studies showed that HIV infection of quiescent T cells could be productive. More specifically, they demonstrated that the viral cDNA was fully reverse transcribed and stably localized in the cytosol. This linear cDNA following T cell activation would then integrate and result in the production of viral progeny [27,30]. Thus, the block was not seen at the early stages of infection such as reverse transcription but later either in nuclear transport or integration [27,30]. However, the key conclusion from these studies was that the block could be easily alleviated at any time after infection with T cell activation, a notion not shared by the studies outlined above [29].

Despite the divergent opinions, this early work clearly demonstrated that the life cycle of HIV in quiescent CD4^+^ T cells was quite distinct from that of activated T cells and warranted further investigation. As technologies evolved, our knowledge was further expanded in regards to the characteristics of the HIV life cycle in quiescent T cells. Studies by Korin et.al utilized a cell cycle progression assay that could assess the levels of both RNA and DNA synthesis and demonstrated that non-dividing T cells can be classified into two categories: (1) cells in the G_o_/G_1a_ phase which is characterized by undetectable levels of DNA and RNA synthesis (truly quiescent) and (2) cells in the G_1b_ phase which is characterized by high levels of RNA expression but not DNA [35]. Following infection of these two sub-populations of non-dividing T cells, it was shown that cells in the G_1b_ stage were susceptible to infection while the truly quiescent G_o_/G_1a_ were resistant [35]. Thus, the data did lend a justification for the disagreement raised in the earlier studies. It would have been possible that the rescue seen after stimulation was due to the fact that G_1b_ phase cells were infected. More importantly, this study underscored the fact that partly activated but non-dividing T cells can be productively infected by HIV and that quiescent T cells are indeed resistant to infection.

Overall these early studies established that HIV replication in quiescent cells is defective. As new and more sensitive technologies developed, groups were able to further dissect and examine in more detail the stages of the viral life cycle that is impacted in quiescent T cells. These studies were more focused on the events leading up to including integration with a growing number interested in post-integration events.

#### Pre-integration blocks to HIV infection in quiescent T cells

A series of studies using more sensitive PCR techniques further supported the opinion that quiescent T cells were resistant to infection and shed some more light on what stages of the HIV life cycle were impacted. The Siliciano group, using a linker-mediated PCR assay, determined that in quiescent T cells reverse transcription occurred at a slower rate, 2–3 days, and produced viral cDNA with a half life of a approximately a day [36]. Despite the formation of full-length viral cDNA, the infection was not productive. In a follow up study, the same group found that the linear non-integrated cDNA was integration competent [37]. Thus, these studies supported and further characterized the presence of labile viral cDNA that was not able to support a productive HIV infection.

Moreover, the development of a sensitive and quantitative assay allowed for the detection of low levels of integration in HIV infected cells [38] and proved to be very useful in the study of HIV infection in quiescent T cells. Using this assay the O’Doherty group demonstrated that quiescent CD4^+^ T cells were infectable by HIV resulting in accumulation of viral cDNA over a three-day period and subsequent integration [39-41]. Furthermore, the authors were able to induce expression of virus following stimulation with IL-7 and anti-CD3/anti-CD28. These studies demonstrated that a productive and latent infection could be established in quiescent cells. However, despite these promising results, the major deficiencies previously seen in quiescent T cells, still persisted and potentially were masked by the use of spinoculation [42] as a method of infection.

Studies done by our group using quantitative real time PCR assays and the integration assay developed by the O’Doherty group analyzed in more detail the kinetics of HIV infection in quiescent CD4 T cells and compared them with that of stimulated T cells [43]. Based on our results, we did not observe any defects on viral entry. However, we did see a significant difference in reverse transcription. Unlike the earlier studies, initiation of reverse transcription was severely decreased (30-fold lower) in quiescent T cells. Interestingly, there was completion of reverse transcription that was delayed by 16 hours. The newly synthesized viral cDNA did integrate in quiescent cells with efficiency similar to that of activated T cells. However, the process was completed 24 hours later than that seen in activated T cells. The integrated provirus found in quiescent T cells did express low levels of multiply spliced viral mRNA, however this did not translate into the expression of detectable viral protein. Interestingly, activation immediately after infection did not rescue this inefficient infection process in quiescent T cells [43]. The results from our studies revealed and pointed to debilitating blocks in the early stages of the viral life cycle as well as delays leading up to viral integration.

#### HIV integration and viral expression defects in quiescent T cells

The finding that there is proviral DNA in quiescent T cells raised the possibility that quiescent T cells can be a reservoir that could support a spreading infection. Integrated virus was previously found in resting cells of HIV infected patients but this was attributed to the infection of previously activated T cells that returned to a resting state [44]. Furthermore, the presence viral mRNA in our studies but the lack of detectable viral protein [43] raised the possibility that HIV integration site selection in quiescent T cells may be distinct from activated ones. Since T cell quiescence is an actively maintained state and HIV preferentially integrates into transcriptionally active units, it would be inferred that a distinct distribution of integration sites could explain our observations. Others and we examined integration site selection in quiescent CD4^+^ T cells [45,46]. Based on our data, integration in both activated and quiescent CD4^+^ T cells occurred in transcriptionally active units such as housekeeping genes that were not affected by cell state [45]. The orientation of integrants between the two cell types was similar as well as the chromosomal locations. Yet, despite the observed similarities, proviral DNA in quiescent cells exhibited higher levels of abnormal LTR-host junctions [45]. Furthermore, we observed higher levels of 2-LTR circles with both normal and abnormal junctions [45]. These patterns suggest that the delays prior to integration had a severe detrimental effect on the ends of the viral cDNA. On the other hand, in the studies by Brady et.al, HIV integration patterns were somewhat different between stimulated and quiescent T cells [46]. HIV integrated in less transcriptionally active regions in quiescent cells when compared to stimulated cells, but the observed differences were modest. Yet, despite the differing conclusions, both studies identified additional potential blocks to HIV infection: (i) LTR attrition that can lead to the integration of defective virions and (ii) integration into transcriptionally repressed regions.

The integration site analysis outlined above however suggested that quiescent T cells might be a source of viral release. To this date, only a handful of studies have examined the post integration events of the HIV life cycle in quiescent cells and in the absence of any stimulation. As quiescent T cells are transcriptionally less active and given the defects in the early stages of infection resulting in mutations of the viral cDNA as well as the potential integration into transcriptionally repressive regions, spontaneous viral release in HIV infected quiescent T cells can also be impaired. Recently studies using the SIV rhesus macaque model suggested that infected resting T cells can spontaneously release virions [47]. However, the transcriptional state of these cells was not fully examined. Our data as well as recent work have shown that multiply spliced *tat/rev* mRNA are lower in HIV infected quiescent and resting CD4 T cells [43,48-51]. This coupled with data from HIV patients on HAART that show elevated levels of unspliced viral mRNA compared to spliced would suggest that defects in splicing can impact the release of virions from quiescent T cells [48,52-54]. Furthermore, low levels of multiply spliced HIV RNA would result in lower levels of Tat protein as it has been shown to play a crucial role in transcriptional elongation [55-62] and recently in RNA splicing [63]. Such an outcome could have detrimental effects in the generation of higher levels of multiply spliced viral RNA. Yet, even if there is production of adequate levels of multiply spliced HIV RNA in quiescent T cells, this is further blocked by reduced nuclear export. This is due to the low levels of the polypyrimidine tract binding protein (PTB) in resting T cells. Low levels of PTB results in nuclear retention of multiply spliced viral RNA thus limiting the production of virions [49,51]. Despite these observed post-integration defects, recent work by Pace and colleagues demonstrated that there is observable but low Gag expression in HIV infected resting T cells [50]. However, this expression of Gag could not support a spreading infection, as the levels of Env protein were very low.

### Restriction factors

While the above studies identified and further refined the stages of HIV life cycle impacted in quiescent T cells, they did not address the mechanisms behind the block. As quiescent T cells are characterized by low transcriptional and metabolic activity, it was reasonable to infer that the lack of cellular substrates or raw materials can have a detrimental effect on viral replication. While pretreatment of quiescent T cells with nucleosides improved reverse transcription in these cells, it failed to rescue infection [64,65]. This suggested that the presence of inhibitory factors or the absence of other supportive processes were responsible for this phenotype.

**Figure 1:**
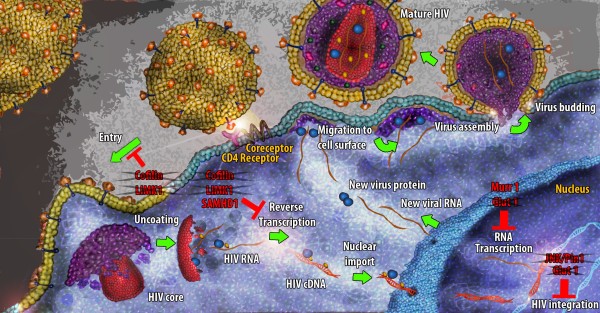
**The HIV life cycle in quiescent CD4**^**+ **^**T cells.** The illustration outlines the major steps in HIV life cycle and the protein factors that are implicated in the observed block. The crossed proteins comprise factors whose lack of expression potentially ameliorates HIV infection.

A number of restriction factors against HIV-1 have been identified over the years such as APOBEC3G [66-80], TRIM5 [81-94], tetherin [95-105], MOV10 [106-109] and recently micro RNAs [110-114]. However, the focus of this review will be on the restriction factors uniquely identified in quiescent CD4^+^ T cells that may be responsible for the observed block to HIV-1 infection (Figure 1).

a. Murr1

Murr1 is involved in copper regulation and inhibits NFκB activity. This inhibition is mediated by blocking proteosomal degradation of IκB resulting in decreased NFκB activity [115]. Studies by Ganesh and colleagues found that the protein is highly expressed in T cells [115]. This in conjunction with the role of NFκB in HIV expression made this a strong candidate for a host restriction factor. Through siRNA-mediated knockdown, the authors demonstrated that downregulation of Murr1 resulted in increased Gag expression suggesting the Murr1 may regulate HIV infection in quiescent CD4^+^ T cells. However, the method of siRNA delivery, nucelofection, even though it did not perturb the activation state of quiescent cells (based on T cell activation marker expression CD25, CD69 and HLA-DR), it may have facilitated infection. While these studies were quite interesting, there was no follow-up work performed to further elucidate the role of this protein.

b. JNK and Pin1

Recent studies highlighted the lack of a cellular protein rather than the presence of a restriction factor as a potential block to HIV infection in quiescent T cells. More specifically, c-Jun N-terminal kinase (JNK) phosphorylates viral integrase, which in turn interacts with the peptidyl prolyl-isomerase enzyme Pin1 causing a conformational change in integrase [116]. This combined effect increases the stability of integrase allowing for viral integration to occur. In these studies quiescent T cells were found not to express JNK, thus ameriolating the role of Pin1 in facilitating HIV intgration [116]. These results lend support to earlier studies demonstrating the presence of preintegrated viral cDNA in resting cells that can act as an inducible reservoir [27,30]. However, these studies did not address the major defects identified by us and others in the early stages of the HIV life cycle as well as the fact that the efficiency of HIV integration in quiescent cells is similar to that of activated cells [39,43,45,46].

c. Glut1

Glut1 has recently been implicated as a potential cellular factor that could facilitate HIV infection. Like JNK and Pin1, the absence of this protein seems to impact HIV infection [117]. Interestingly, its role is linked to the metabolic processes of T cells. More specifically, Glut1 is a major glucose transporter found in both mature T cells and thymocytes [117]. Protein expression is upregulated by IL-7 treatment or conventional T cell activation. When Glut expression was knockdown in activated T cells, it resulted in decreased HIV infection of these cells[117]. Expression levels of the protein were further correlated with permissiveness to HIV infection as double positive thymocytes expressing high levels of Glut1 were more likely to be infected by HIV than their low expressing counterparts [117]. This study is quite intriguing as it is the first one linking cell metabolism to HIV replication. depolymerization factor

d. Cytoskeleton

The cytoskeleton has been shown to have a key role in HIV replication [118]. The cell structure plays a key role in cell shape, motility, organelle organization and intracellular trafficking [118]. This section involves multiple factors that have been recently identified to facilitate or block HIV infection. Early work showed that the HIV reverse transcription complex interacted with actin and disruption of the interaction resulted in blocking infection [119]. These studies suggested that the cytoskeleton was crucial for a productive HIV infection. Subsequent studies explored the molecular mechanisms of this phenomenon. More specifically, Yoder and colleagues showed that cross linking of CXCR4, one of the co-receptors for the virus, results in activation of cofilin, an actin that allows for HIV to rearrange actin and, consequently, facilitate infection [120]. This was further supported by patient studies showing that resting T cells isolated from HIV infected individuals had elevated levels of active cofilin thus facilitating the spread of infection [121]. In addition to CXCR4, crosslinking of CCR7, CXCR3, and CCR6 have been shown to activate cofilin and mediate the establishment of a latent HIV infection in resting T cells [122]. However, cofilin is not the sole factor involved in the interplay between HIV and actin. LIM domain kinase 1 (LIMK1), which phosphorylates cofilin and inactivates it, becomes activated following cross-linking of gp120 and CXCR4 [123]. This leads to actin polymerization and stabilization of the CD4/CXCR4 cluster allowing for efficient viral entry and uncoating. The stable complex then activates cofilin to further facilitate infection. This pathway was recently shown to be disrupted by the N-terminal fragment of Slit2 a secreted glycoprotein and reversed the HIV mediated changes in actin thus inhibiting infection [124]. These studies underscore the importance of cytoskeleton in HIV infection and have become an exciting area of HIV research as they can lead to the development of new therapies against the virus.

e. SAMDH1

The Sterile Alpha Motif (SAM) domain and HD domain-containing protein 1 (SAMHD1) has been recently identified as a potential restriction factor in quiescent T cells. SAMHD1, like APOBEC3G, seems to target the early stages of the HIV life cycle more specifically reverse transcription. SAMHD1 is mutated in a subset of patients suffering from the Aicardi-Goutieres syndrome (AGS), an early-onset encephalopathy that mimics a congenital infection and is associated with increased levels of IFN-α production [125]. Studies suggested that the protein may be involved in negatively regulating innate immune responses [125]. With regards to HIV restriction, studies showed that SAMHD1 mediated the restriction to HIV infection in dendritic cells and monocytes [126-128]. The observed restriction of SAMHD1 was alleviated by the lentiviral protein Vpx, which is expressed in SIV[127,129]. When Vpx, a relative of Vpr the viral accessory protein expressed in HIV-1, was introduced into macrophages and monocyte derived dendritic cells, it significantly enhanced their infection by HIV [130-132]. Additional studies revealed that SAMHD1 is a strong dGTP triphosphohydrolase, thus impacting total nucleotide pools in cells [133,134]. By depleting these pools SAMHD1 inhibits reverse transcription thus restricting HIV replication [135].

While a number of studies employed gene knockdown to further elucidate the role of SAMHD1 and other restriction factors in HIV infection, the use of cell samples from AGS patients has proven particularly beneficial as it eliminated the variable of cell manipulation. Monocytes and dendritic cells from AGS patients were susceptible to HIV infection [127,128]. With respect to quiescent T cells, two studies have independently shown that the protein is abundantly expressed in them [136,137]. Both the SAMDH1 depleted and AGS patient derived CD4 T cells demonstrated improved HIV infection due to increased reverse transcription [136,137]. However, the expression of viral progeny was still defective in quiescent cells as suggested by both studies. In addition, even though SAMHD1 is also highly expressed in activated T cells, its inhibitory effects are only seen in quiescent T cells [127,135,136]. Thus, the combination of both limiting endogenous pools of nucleotides in quiescent T cells and the presence of SAMHD1 have a combined inhibitory effect on viral replication.

As the field further explores the role of SAMHD1 it is clear that the protein limits the available nucleotide pools in quiescent cells thus restricting efficient reverse transcription. However, as previous studies have shown, a mere addition of nucleosides while improving reverse transcription does not remedy the block seen in quiescent T cells [64,65].

## Conclusions

In conclusion, the mechanisms and/or cellular factors mediating the block in the HIV infection of quiescent CD4 T cell are not fully understood yet. While a number of cellular factors have been implicated, it is clear that the blocking effect to HIV infection is mediated by multiple events due to the physiology of quiescent T cells. Cellular size, transcriptional and metabolic activities are all important cell functions that are used by intracellular parasites such as viruses to successfully infect and replicate into the host cells.

Based on the early and subsequent work, the characterization of the HIV life cycle in quiescent T cells strongly indicate that the major impact to infection occurs very early, immediately following viral entry at the initiation of reverse transcription. While limited raw materials such as nucleotides impacted by both the nature of quiescent cells and SAMHD1 can result in decreased levels of reverse transcription, it is clear that downstream events prior to integration or even at integration are quite important. In addition, another process that is widely bypassed due to technical challenges, uncoating can be impacted in quiescent cells and be detrimental to infection [138,139].

Therefore, further studies are needed to understand the block in quiescent T cells. To this date, based on what we know and the nature of cellular factors identified, it is not clear how the mechanisms of resistance in quiescent cells can translate into future therapies. Nevertheless, these studies will allow us to better understand the relationship between HIV and it various target cells which can ultimately can lead to more effective interventions.

## Competing interests

The authors declare that they have no competing interests.

## Authors’ contributions

JAZ, SGK and DNV wrote and edited the manuscript; SGK prepared the manuscript figure. All authors read and approved the final manuscript.
